# Mesenchymal Hamartoma Mimicking Hepatoblastoma

**Published:** 2014-05-01

**Authors:** A. Bahador, B. Geramizadeh, M. Rezazadehkermani, S. Moslemi

**Affiliations:** 1 *Departments of Pediatrics Surgery, Shiraz University of Medical Sciences, Shiraz, Iran*; 2 *Departments of Pathology, Shiraz University of Medical Sciences, Shiraz, Iran*; 3 *Departments of General Surgery, Shiraz University of Medical Sciences, Shiraz, Iran*

**Keywords:** Liver tumor, Mesenchymal hamartoma, Hepatoblastoma, Alpha-fetoprotein

## Abstract

Mesenchymal hamartoma and hepatoblastoma are common causes of hepatic masses in pediatric population; they have similar radiologic and pathologic features. Herein, we present a case of mesenchymal hamartoma that was preoperatively diagnosed as hepatoblastoma. The mass was completely resected instead of being treated with preoperative chemotherapy. Postoperative pathological evaluation revealed mesenchymal hamartoma with free margins; the patient incidentally received the standard treatment. If we would have measured serum AFP in our patient, we could make the correct diagnosis preoperatively, because AFP increases largely in hepatoblastoma. When suspicious exists, serum AFP is a good guide in differentiating hepatoblastoma from mesenchymal hamartoma.

## INTRODUCTION

Mesenchymal hamartoma is the second most common benign tumor of liver in childhood. Most of these tumors are large multicystic masses that usually diagnosed before the third year of life mostly in boys. Mesenchymal hamartoma may present with various signs and symptoms like anorexia, vomiting, abdominal distension, and poor weight gain, but pain is rarely a dominant feature. Laboratory studies usually reveal normal liver function tests with moderately elevated serum α-fetoprotein (AFP). Ultrasound, CT, and MRI demonstrate a multiloculated cystic tumor with variable amount of solid tissue[1].

Hepatoblastoma is the most common malignant tumor of childhood which is more frequent in boys. It is usually diagnosed in young children with a peak incidence in newborns [2]. It usually presents with an enlarging abdominal mass mostly involving the right liver lobe. Anorexia, weight loss, and pain are less common symptoms. Serum AFP is almost always elevated and other liver function tests are usually normal. Radiologic evaluations demonstrate a focal or multifocal solid tumor with calcification in 40%–50% of cases, which is not essential for diagnosis [3].

In most patients, diagnosis of mesenchymal hamartoma is suggested by imaging and confirmed with histologic examination. Aspiration cytology rarely enables a definite diagnosis and due to its inability to ruling out hepatoblastoma or other malignant mesenchymal tumors, it has limited diagnostic value [1].

The main treatment for mesenchymal hamartoma is complete excision of the mass, whereas the first line for the treatment of hepatoblastoma may be chemotherapy [3], thus, making a preoperative differential diagnosis between these two conditions is paramount.

Herein, we present a case of liver tumor that was primarily diagnosed as hepatoblastoma, but it was found to be really mesenchymal hamartoma, postoperatively. 

## CASE PRESENTATION

**Figure1:**
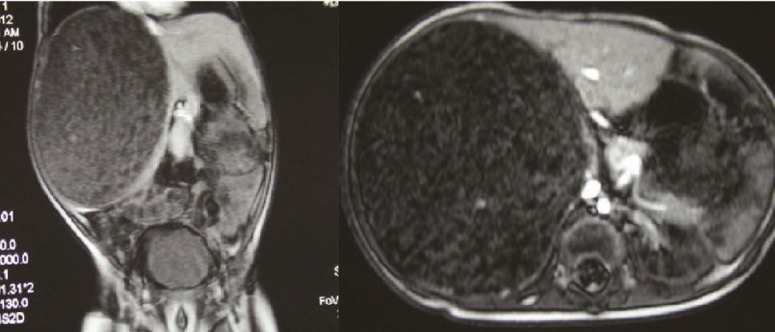
Abdomenopelvic MRI (sagittal and coronal) reveals a large well-defined heterogeneous mass measured 12×10 cm in the right lobe of liver causing displacement of and compression on the adjacent vessels and right kidney, in favor of hepatoblastoma

A 13-month-old boy, a known case of thalassemia major, was referred to our center from Jiroft, South-east of Iran, because of an abdominal mass diagnosed two months before. He had history of jaundice and diarrhea during this period. Abdominal ultrasonography prior to his admission, showed a 114-mm hyperechoic mass in the right liver lobe. Abdominal MRI showed a large well-defined heterogeneous mass 10×12 cm in diameter in the right liver lobe; the mass displaced the right kidney and compressed the adjacent vessels (Fig 1). Both radiologist who reported the ultrasonography and MRI, suggested the diagnosis of hepatoblastoma. 

Open liver biopsy, done for the patient 20 days before his referral, was also reported as hepatoblastoma. Patient was referred to Pediatric Oncology service for receiving preoperative chemotherapy, however, because of some personal problems, parents of the patient did not with the preoperative chemotherapy. Therefore, we decided to operate the patient because the mass seemed to be resectable in MRI.

The abdominal cavity was entered via a Chevron incision. The standard abdominal exploration was done. No sign of peritoneal seeding or involvement of adjacent organs was observed. After releasing the falciform, right triangular, and right coronary ligaments, the right liver lobe was exposed. Right hepatic artery, and middle and right hepatic veins were ligated. Bleeders were controlled. After the extension of tumor was evaluated, right extended hepatectomy was performed.

Gross pathology report described tumor as a well-defined creamy green mass with measured at 12×11×8 cm; the margins were grossly free of tumor. Microscopic evaluation was in favor of mesenchymal hamartoma. The patient was discharged from hospital in good condition.

## DISCUSSION

Our patient was found to have liver mesenchymal hamartoma that resembled hepatoblastoma preoperatively. A similar report was also published from Turkey in 2008 [4]. The patient presented in Turkish report was an 11-month-old boy with liver mass, elevated serum AFP and a needle biopsy in favor of hepatoblastoma. The patient underwent preoperative chemotherapy after which the serum AFP and tumor size decreased. Nonetheless, after right hepatectomy, pathological examination revealed the tumor was in fact mesenchymal hamartoma.

In the differential list of a pediatric liver mass with various amount of solid tissue and cystic components in radiologic evaluation, are hepatoblastoma, embryonal sarcoma, hemangioma, infantile hemangioendothelioma, hepatic hydatid cyst, and mesenchymal hamartoma [5]. There are different histologic types defined for hepatoblastoma, among which the mixed epithelial mesenchymal hepatoblastoma resembles mesenchymal hamartoma. This particular type of hepatoblastoma can be composed of relatively bland appearing hepatocyte with spindle/stellate mesenchyme, which is seen in mesenchymal hamartoma too [5].

The treatment strategy for hepatoblastoma is combined chemotherapy and surgery.[6] However, if at initial laparotomy, the tumor seems resectable, reasonably safe attempts should be made to excise the tumor [3]. Mesenchymal hamartoma is treated with complete resection. Liver transplantation is proposed for unresectable tumors, and chemotherapy has limited value in treating mesenchymal hamartoma [1].

If we would have measured serum AFP in our patient, we could make the correct diagnosis preoperatively, because AFP increases largely in hepatoblastoma. When suspicious exists, serum AFP is a good guide in differentiating hepatoblastoma from mesenchymal hamartoma.
